# Targeting Brain Cholesterol Homeostasis in Alzheimer's Disease: Mechanisms and Therapeutic Perspectives

**DOI:** 10.1111/jnc.70492

**Published:** 2026-06-12

**Authors:** Myuri Ruthirakuhan, Ameer Y. Taha, Walter Swardfager

**Affiliations:** ^1^ Hurvitz Brain Sciences Program Sunnybrook Research Institute Toronto Ontario Canada; ^2^ Department of Food Science and Technology, College of Agriculture and Environmental Sciences University of California Davis California USA; ^3^ Department of Pharmacology and Toxicology University of Toronto Toronto Ontario Canada

## Abstract

Cholesterol is a fundamental component of the central nervous system, supporting myelin integrity, synaptic structure, membrane organization, and neuronal signaling. Because the brain is largely isolated from peripheral cholesterol pools, tight regulation of brain cholesterol homeostasis is required to sustain neuronal and glial function across the lifespan. Growing evidence indicates that disruption of this balance is not merely a downstream consequence of neurodegeneration, but an upstream contributor to Alzheimer's disease (AD) pathogenesis. Altered brain cholesterol homeostasis has been linked to amyloidogenic processing, tau pathology, neuroinflammation, synaptic dysfunction, and cerebrovascular injury. This review synthesizes current evidence showing how multiple converging stressors, including peripheral hypercholesterolemia, neurodegeneration, oxidative stress, and inflammatory signaling, perturb brain cholesterol regulation. These drivers disrupt the coordinated processes of cholesterol synthesis, metabolism, and transport, shifting the system from tightly regulated sterol flux toward impaired clearance, abnormal lipid distribution, and membrane instability. Such disturbances remodel membrane lipid composition, alter lipid raft organization, and impair glial–neuronal lipid coupling, thereby accelerating amyloid‐β production, tau‐related vulnerability, innate immune activation, and neurovascular dysfunction. Finally, we provide an overview of therapeutic strategies aimed at restoring cholesterol balance, and highlight the potential of integrated, multi‐target strategies to complement amyloid‐ and tau‐directed therapies. By clarifying how disruptions in brain cholesterol homeostasis link systemic and central stressors to AD pathology, this review identifies cholesterol regulation as a critical, upstream axis for therapeutic intervention and disease prevention.

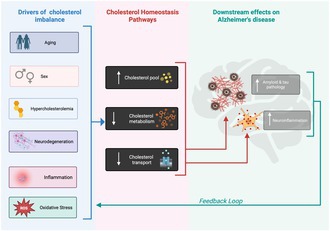

## Graphical abstract

Brain cholesterol homeostasis is tightly regulated and largely independent of peripheral cholesterol. This review proposes a unifying framework in which aging, sex, and related biological stressors, including hypercholesterolemia, neurodegeneration, inflammation, and oxidative stress, converge to disrupt cholesterol homeostasis in the brain. Impairments in cholesterol metabolism and transport lead to lipid accumulation and promote amyloid and tau pathology, neuroinflammation, and neurodegeneration, forming a self‐amplifying feedback loop in Alzheimer's disease.

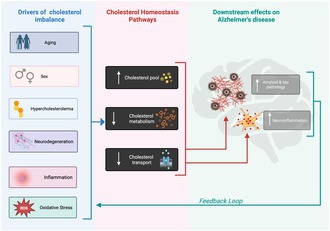

**Abbreviations**
24S‐HOC: 24S‐hydroxycholesterol
25‐HOC: 25‐hydroxycholesterol
27‐HOC: 27‐hydroxycholesterol
7‐DHC: 7‐dehydrocholesterol
7‐HOCA: 7a‐hydroxy‐3‐oxo‐4‐cholestenoic acid
7‐KC: 7‐ketocholesterol
7α/7β‐HOC: 7α/7β‐hydroxycholesterol
ABC transporters: ATP‐binding cassette transporters
AD: Alzheimer's disease
APOE4: Apolipoprotein E4
APP: Amyloid precursor protein
BBB: Blood–brain barrier
CH25H: 25‐hydroxylase
*CLU*
: Clusterin
CNS: Central nervous system
CoA: Coenzyme A
CYP27A1: Cholesterol 27‐hydroxylase
CYP46A1: Cholesterol 24‐hydroxylase
DHCR24: 24‐dehydrocholesterol reductase
DHCR7: 7‐dehydrocholesterol reductase
ER: Endoplasmic reticulum
FDFT1: Squalene synthase
FPP: Farnesyl pyrophosphate
GPP: Geranyl pyrophosphate
HMG‐CoA: 3‐hydroxy‐3‐methylglutaryl‐CoA
HMGCR: 3‐hydroxy‐3‐methylglutaryl‐CoA reductase
INSIG: Insulin‐induced gene
LDL: Low‐density lipoprotein
LRP1: Lipoprotein receptor‐related protein 1
LXR: Liver X receptor
mEH: Microsomal epoxide hydrolase
NLRP3: NOD‐, LRR‐ and pyrin domain containing protein 3
PUFAs: Polyunsaturated fatty acids
PVS: Perivascular space
ROS: Reactive oxygen species
SCAP: SREBP cleavage‐activating protein
sEH: Soluble epoxide hydrolase
SQLE: Squalene epoxidase
SQS/FDFT1: Squalene synthase
SREBP: Sterol Regulatory Element‐Binding Protein
TLR: Toll‐like receptors
TREM2: Triggering Receptor Expressed on Myeloid Cells 2
WMH: White matter hyperintensities

## 1 Introduction

Cholesterol is an essential lipid in the central nervous system (CNS), where it serves as a critical structural and functional component of neuronal membranes. Although the brain represents only ~2% of the body's total weight, it contains approximately 20% of the body's total cholesterol, reflecting the brain's unique demands for cholesterol in maintaining cellular and neuronal integrity and function (Dietschy and Turley 2001). Approximately 70%–80% of brain cholesterol is located in myelin sheaths, while the remainder resides in plasma membranes of neurons and glial cells (Björkhem and Meaney 2004). Within plasma membranes, cholesterol supports synapse formation, dendritic spine maturation, and neurotransmitter signaling. Through its role in assembling lipid rafts, which includes dynamic membrane domains that organize signaling and trafficking machinery, cholesterol influences amyloid precursor protein (APP) processing (Pantelopulos et al. 2024; Hicks et al. 2012), thereby linking membrane composition to Alzheimer's disease (AD) pathogenesis. These functions highlight why even modest disturbances in brain cholesterol regulation can have widespread consequences for neuronal signaling, glial function, and neurovascular integrity.

Given these critical roles, tight regulation of brain cholesterol homeostasis is essential. Emerging evidence indicates that cholesterol dysregulation contributes directly to AD pathology by impacting amyloid‐beta production (Pantelopulos et al. 2024), tau phosphorylation (Wang and Zhang 2022), and neuroinflammation (de Dios and Abadin 2023). These disturbances do not arise from a single cause but rather reflect the cumulative impact of biological stressors that disrupt cholesterol homeostasis across the lifespan. Aging and related biological stressors, including hypercholesterolemia, neurodegeneration, oxidative stress, and inflammatory signaling, can each perturb different aspects of cholesterol synthesis, metabolism, and transport within the brain (Morgan et al. 2016; Owen and Sultana 2010; Itri et al. 2014; Romero et al. 2024). Central cholesterol imbalance therefore represents an upstream mechanism driving the molecular cascade that leads to synaptic loss, neurodegeneration, and AD pathogenesis.

Of relevance, the apolipoprotein E4 (APOE4) allele, which is the strongest genetic risk factor for late‐onset AD (Raulin and Doss 2022; Strittmatter and Saunders 1993), alters cholesterol trafficking, lipidation, and transport within the brain, promoting AD‐related neurodegeneration and underscoring the importance of cholesterol homeostasis in AD pathogenesis. Notably, the impact of APOE4 on AD risk and disease progression differs between females and males (Ungar et al. 2014), highlighting the importance of considering sex as a biological variable when examining lipid metabolism and vulnerability to neurodegeneration.

Despite intense research efforts, current therapies for AD disease modification, such as anti‐amyloid agents, have demonstrated modest efficacy, emphasizing the urgent need to explore complementary biological pathways that underlie AD disease onset and progression, such as cholesterol homeostatic pathways. Although cholesterol imbalance is increasingly recognized as a key component of neurodegeneration, therapeutic strategies directly targeting cholesterol pathways remain limited, in part because the biological drivers that destabilize cholesterol homeostasis across aging and disease remain poorly defined.

**FIGURE 1:**
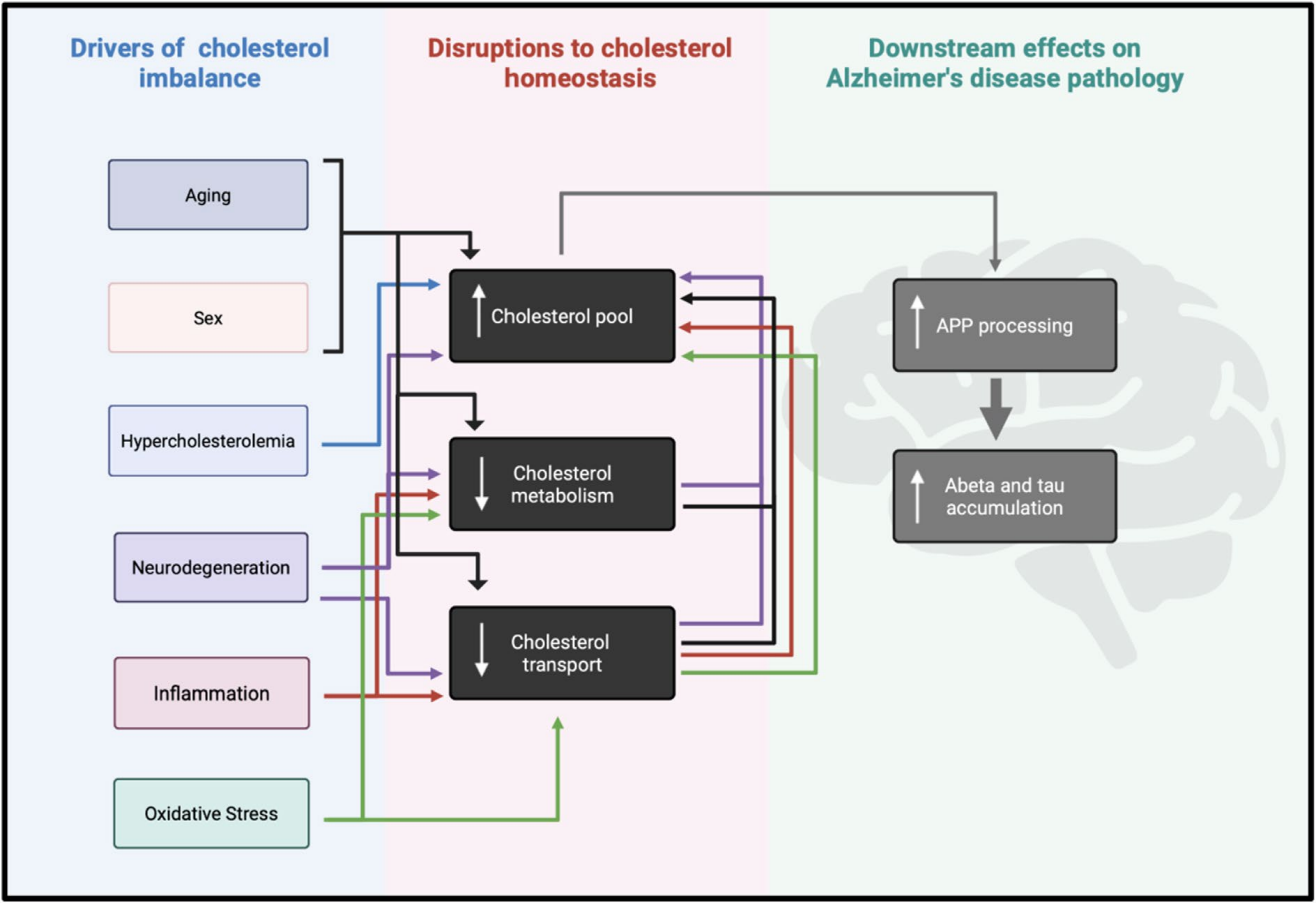
Conceptual framework illustrating drivers of cholesterol dyshomeostasis. Aging and other biological stressors disrupt cholesterol synthesis, metabolism, and transport, resulting in negative impacts on AD pathogenesis. Although depicted directionally for conceptual clarity, amyloid and tau pathology may further exacerbate cholesterol dyshomeostasis through downstream effects on neuronal function, oxidative stress, and inflammation.

In this review, we summarize current evidence on cholesterol synthesis, metabolism, transport, and intracellular handling in the CNS, integrating how each pathway is perturbed by biological stressors that arise during aging and AD. Our framework proposes that aging, hypercholesterolemia, neurodegeneration, oxidative stress, and inflammation converge on the core regulatory mechanisms of cholesterol homeostasis, resulting in impaired synthesis, altered turnover, defective transport, and abnormal lipid distribution (Figure 1). These disruptions, in turn, reshape membrane architecture, destabilize glial–neuronal lipid coupling, and promote downstream consequences relevant to AD, including amyloidogenic APP processing, tau vulnerability, neuroinflammation, myelin and white matter injury, and neurovascular dysfunction. By organizing the literature around this driver–pathway–consequence model, we aim to clarify how cholesterol dysregulation links systemic and central stressors to AD pathogenesis and to identify upstream points for therapeutic intervention.

## 2 Key Pathways in Cholesterol Homeostasis

**FIGURE 2:**
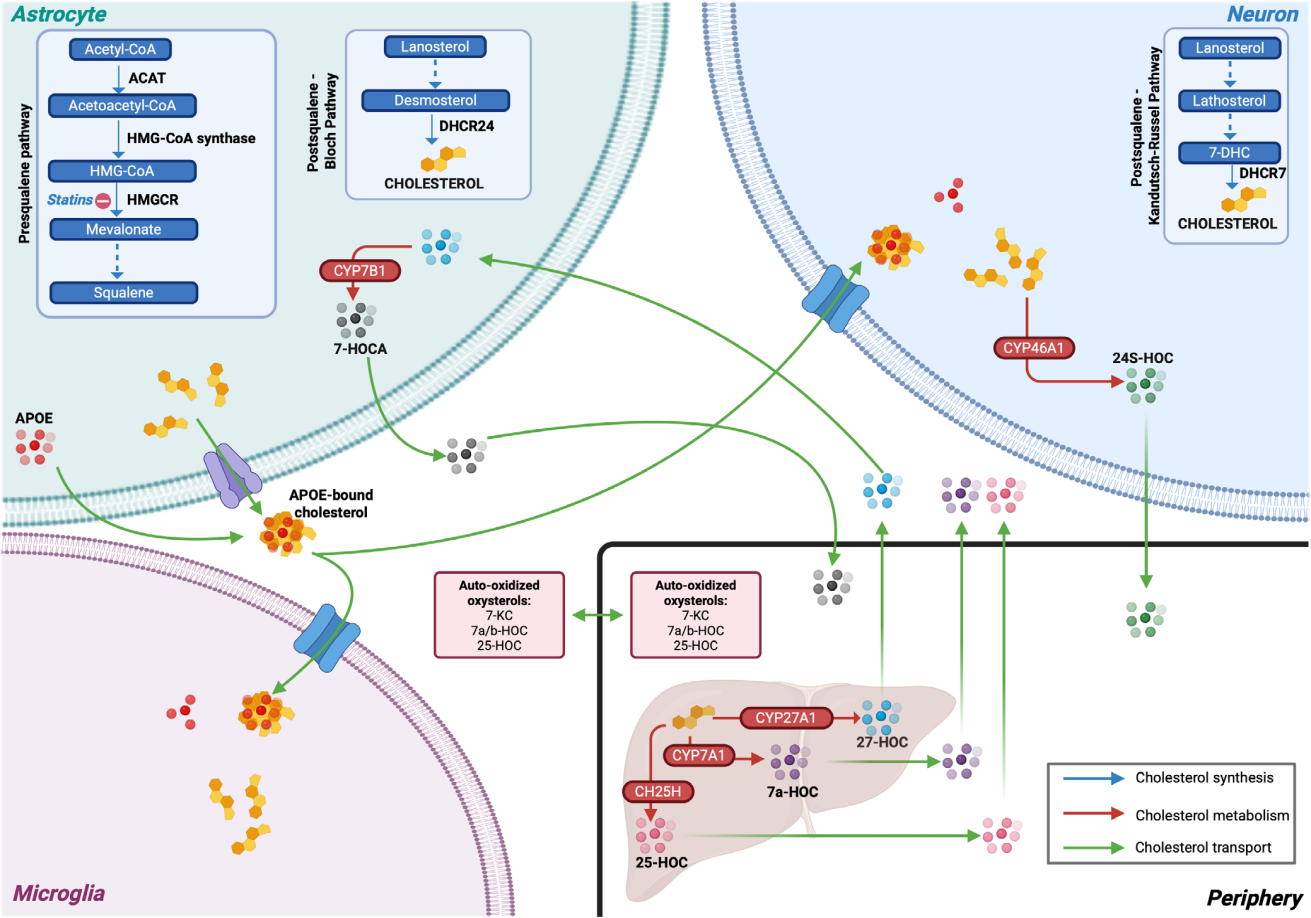
Integrated pathways of cholesterol synthesis, metabolism, and transport. Cholesterol homeostasis in the CNS is maintained through coordinated interactions between astrocytes, neurons, and microglia. Astrocytes synthesize cholesterol de novo and export it via APOE‐containing lipoproteins to support neuronal function. Neurons, which have limited synthetic capacity, rely on astrocyte‐derived cholesterol and mediate its elimination through CYP46A1‐dependent conversion to 24S‐HOC, which exits the brain across the blood–brain barrier. Peripheral cholesterol metabolism generates 27‐HOC, which enters the brain and is further metabolized by CYP7B1, linking peripheral and central sterol pools. Additional oxysterols arise through enzymatic and auto‐oxidative pathways. These integrated processes regulate cholesterol distribution, turnover, and cross‐compartment signaling in the healthy brain.

Approximately 99% of cholesterol in the brain is present in the free (unbound) form; only a small amount is bound to fatty acids in the form of cholesteryl esters (Björkhem and Meaney 2004). Free cholesterol homeostasis in the brain is maintained through a coordinated network of synthesis, metabolism, and transport, the three core pathways that collectively regulate cholesterol production, turnover, and cellular distribution across neurons, astrocytes, and oligodendrocytes (Figure 2). These pathways are normally subject to tight feedback control, ensuring that cholesterol levels remain within a narrow physiological range required for membrane structure, synaptic function, and myelin stability.

### 2.1 Cholesterol Synthesis

The brain relies primarily on local de novo cholesterol synthesis due to the impermeability of the blood–brain barrier (BBB) to lipoprotein‐bound cholesterol (Björkhem and Meaney 2004). Astrocytes are the main site of synthesis, which then supply cholesterol to neurons for axonal growth and synaptogenesis, while neuronal synthesis is modest and declines with age (Zhang and Liu 2015; Pfrieger 2003).

Brain cholesterol synthesis can be divided into two stages: (1) the presqualene (mevalonate) pathway, and (2) the post‐squalene pathway. The presqualene pathway, which primarily occurs in the astrocytes, encompasses a series of enzymatic reactions that convert acetyl‐coenzyme A (CoA) to squalene. This pathway begins with the condensation of acetyl‐CoA to acetoacetyl‐CoA, followed by the synthesis of 3‐hydroxy‐3‐methylglutaryl‐CoA (HMG‐CoA) by HMG‐CoA synthase, and reduction of HMG‐CoA to mevalonate by 3‐hydroxy‐3‐methylglutaryl‐CoA reductase (HMGCR), which is the rate limiting enzyme and primary target of pharmacological intervention. Mevalonate is then converted to isopentenyl pyrophosphate (IPP) and subsequently geranyl and farnesyl pyrophosphate (GPP/FPP). The terminal step of this pathway is the formation of squalene by squalene synthase (FDFT1) (Moutinho et al. 2017). The presqualene pathway primarily occurs in the endoplasmic reticulum (ER), where key enzymes such as HMGCR and squalene synthase are membrane bound (Stamellos and Shackelford 1993; Mohamed et al. 2015; Jo and DeBose‐Boyd 2022). However, earlier steps, including acetyl‐CoA condensation and mevalonate phosphorylation, occur in the cytosol (Mohamed et al. 2015).

Following squalene formation, cholesterol synthesis proceeds through the post‐squalene pathway, which bifurcates into the Bloch and Kandutsch–Russell pathways. Both pathways convert squalene to lanosterol through a series of enzymatic reactions that first start with squalene epoxidase (SQLE), an oxygen‐dependent enzyme. In the Bloch pathway, which predominantly occurs in the astrocytes, lanosterol is converted to desmosterol through multiple intermediates, with 24‐dehydrocholesterol reductase (DHCR24), also known as Seladin‐1, catalyzing the final step to cholesterol. In the Kandutsch–Russell pathway, which predominantly occurs in the neurons (Genaro‐Mattos et al. 2019), lanosterol is converted to lathosterol and 7‐dehydrocholesterol (7‐DHC) through multiple intermediates, with 7‐dehydrocholesterol reductase (DHCR7) catalyzing the final conversion to cholesterol.

**FIGURE 3:**
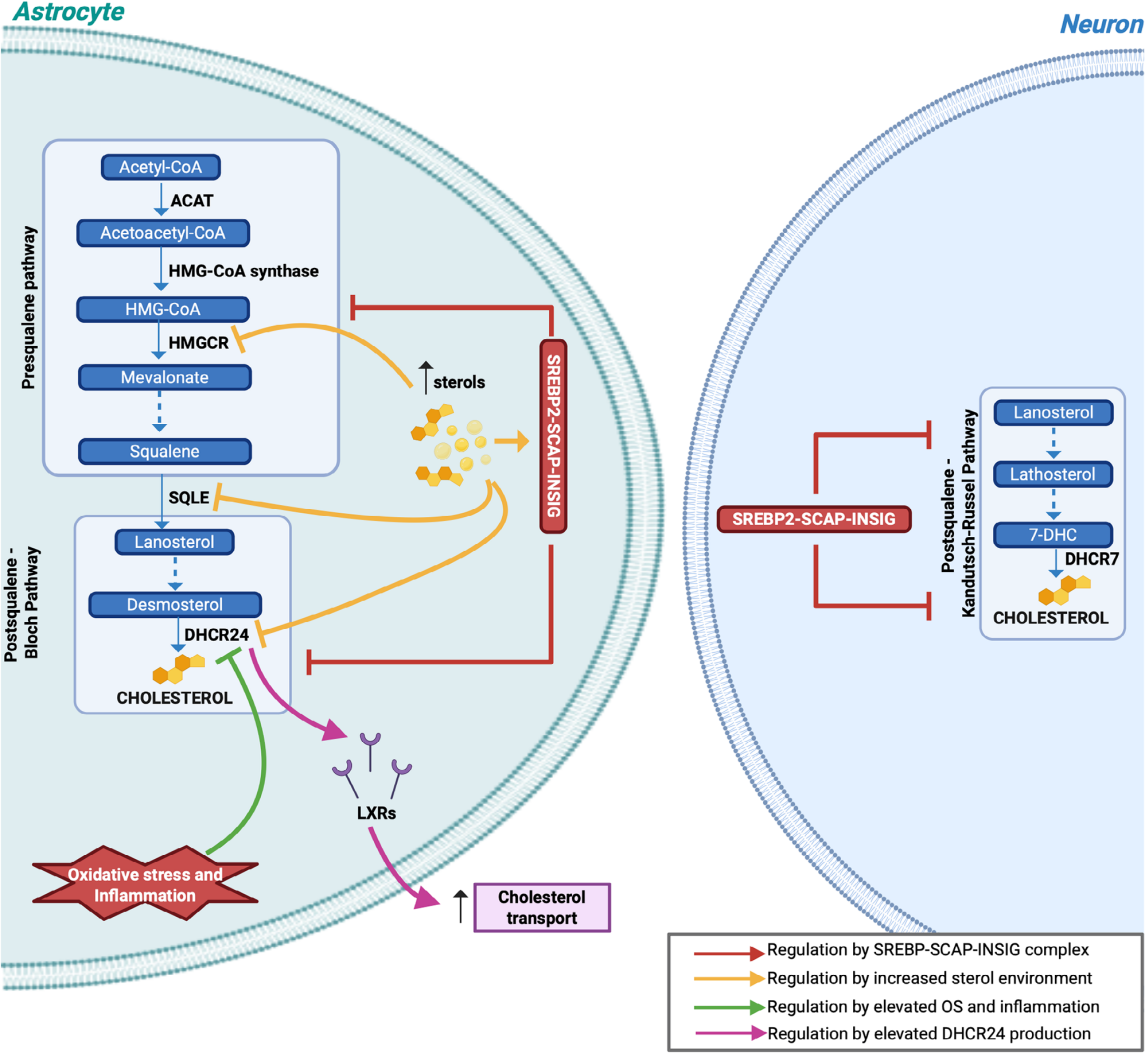
Regulatory mechanisms governing cholesterol synthesis in the CNS. Cholesterol synthesis is controlled through coordinated regulation of the presqualene (mevalonate) and post‐squalene pathways across astrocytes and neurons. Astrocytes are the primary site of synthesis, with HMGCR serving as the rate‐limiting enzyme, while neurons exhibit reduced synthetic capacity and rely on distinct downstream enzymatic steps (e.g., DHCR7). The SREBP2–SCAP–INSIG complex functions as the central sterol‐sensing system, activating transcription of cholesterol synthesis genes under low sterol conditions and suppressing synthesis when sterol levels are elevated. Oxidative stress and inflammation impair key enzymatic steps, while LXR signaling links intracellular sterol levels to cholesterol transport pathways. These integrated regulatory mechanisms ensure that cholesterol synthesis is tightly matched to cellular demand and environmental stress.

Together, the key regulatory checkpoints of cholesterol synthesis include HMGCR (presqualene pathway), and SQLE, DHCR24, and DHCR7 (postsqualene pathway), which coordinate the rate of cholesterol synthesis and adjust it according to the local sterol environment under normal physiological conditions. Upstream, the SREBP2‐SCAP‐INSIG complex is another key regulatory component which is the primary transcriptional switch that controls the expression of pre‐ and postsqualene pathway enzymes (Figure 3). Under normal physiological conditions, sterols bind to SCAP or INSIG, which signals ER retention of SREBP2, downregulating the production of cholesterol synthesis genes (Radhakrishnan et al. 2008). This multi‐level regulation ensures that cholesterol production is efficiently matched to its cellular demand.

### 2.2 Cholesterol Metabolism

Cholesterol metabolism is a critical mechanism for maintaining cholesterol homeostasis and preventing its harmful accumulation. The conversion of cholesterol to oxysterols is its only route for elimination, and this can occur through (i) brain cholesterol metabolism, (ii) peripheral cholesterol metabolism, and (iii) alternative pathways such as auto‐oxidation.

#### 2.2.1 Central Cholesterol Metabolism

Cholesterol in the brain is isolated from peripheral cholesterol pools by the BBB. As lipoprotein‐bound cholesterol is not thought to cross the BBB, its regulation relies on its independent synthesis and metabolism in the brain. In support of this, knockout of key cholesterol transporters does not significantly alter brain cholesterol levels, underscoring the independence of central cholesterol homeostasis from peripheral cholesterol pools (Taha and Chen 2009; Rahman and Taha 2010). The primary route of central cholesterol elimination is through cholesterol 24‐hydroxylase (CYP46A1), which is predominantly expressed in neurons (Ramirez et al. 2008; Popiolek and Izumi 2020). CYP46A1 converts central cholesterol to 24S‐hydroxycholesterol (24S‐HOC), a more hydrophilic oxysterol that can diffuse across the BBB into the periphery, where it is ultimately cleared by the liver (Russell et al. 2009). In addition to serving as the principal route of cholesterol elimination from the brain, 24S‐HOC also functions locally as a bioactive oxysterol that modulates neuronal signaling, glial responses, and sterol‐sensitive transcriptional pathways within the CNS (Sun et al. 2016; Noguchi et al. 2014; Wang et al. 2008). Specifically, in astrocytes and microglia, it regulates lipid metabolism and inflammatory signaling through liver X receptor (LXR)‐dependent transcriptional programs, linking cholesterol turnover to local cellular function (Sun et al. 2016; Abildayeva and Jansen 2006).

#### 2.2.2 Peripheral Cholesterol Metabolism

A second important route for regulating cholesterol homeostasis between the brain and periphery involves peripheral cholesterol metabolism via cholesterol 27‐hydroxylase (CYP27A1) which is primarily located in the liver (Russell 2003). CYP27A1 generates 27‐hydroxycholesterol (27‐HOC) which can passively cross the BBB using concentration gradients (Heverin and Meaney 2005), and can therefore act as a peripheral cholesterol signal to the brain. Once in the CNS, 27‐HOC is further metabolized by CYP7B1 to 7a‐hydroxy‐3‐oxo‐4‐cholestenoic acid (7‐HOCA), a more polar oxysterol that can passively exit the brain to the periphery for hepatic elimination (Meaney and Heverin 2007). Therefore, peripheral 7‐HOCA has been used as an indicator of net 27‐HOC flux through the CNS (Saeed and Floris 2014).

#### 2.2.3 Alternative Metabolic Pathways

In addition to the major central and peripheral enzymatic pathways, cholesterol can also be converted to oxysterols through (i) auto‐oxidation and (ii) enzymatic production. Under normal physiological conditions, these pathways contribute minimally to overall cholesterol turnover.

- Auto‐oxidation: Cholesterol undergoes low‐level auto‐oxidation driven by background reactive oxygen species (ROS) and normal metabolic activity. This produces oxysterols such as 7‐ketocholesterol (7‐KC) and 7α/7β‐hydroxycholesterol (7α/7β‐HOC) (Zerbinati and Iuliano 2017). In healthy conditions, their formation is limited and balanced by efficient cellular detoxification, esterification, and export mechanisms.
- Enzymatic production: Some oxysterols are produced enzymatically in response to physiological immune signaling. 25‐hydroxycholesterol (25‐HOC) is generated by cholesterol 25‐hydroxylase (CH25H), a tightly regulated enzyme expressed in macrophages, microglia, and other immune cells (Zerbinati and Iuliano 2017; Zhao et al. 2020; Cashikar and Toral‐Rios 2023). Even under basal immune tone, CH25H contributes small amounts of 25‐HOC, which plays homeostatic roles in lipid metabolism and innate immune modulation (e.g., tonic regulation of SREBP and LXR pathways). Other enzymes, including CYP7A1 and CYP7B1, can generate low levels of 7α‐HOC as part of normal bile acid–related and extrahepatic sterol metabolism (Russell 2003).
  Together, these central, peripheral, and alternative metabolic pathways ensure the efficient turnover of cholesterol and prevent its harmful accumulation in the brain.

### 2.3 Intercellular Cholesterol Transport

While central and peripheral metabolism and the controlled oxidation of cholesterol are essential for maintaining overall cholesterol homeostasis and preventing free cholesterol accumulation, the brain also relies on intercellular transport of free cholesterol to meet the high metabolic and structural demands of neurons. Since neurons reduce their own ability to synthesize cholesterol with age, they rely on astrocytes for a continuous supply of cholesterol necessary for membrane synthesis, synapse formation, and repair. This distribution is accomplished through coordinated mechanisms of cholesterol efflux, redistribution, and influx among astrocytes, neurons, microglia, and oligodendrocytes. Although less frequently emphasized in AD‐focused models, oligodendrocytes and myelin‐associated lipid turnover represent an important component of brain cholesterol homeostasis, particularly in the aging brain where remyelination efficiency declines (Huang et al. 2025).

#### 2.3.1 Cholesterol Efflux

Efflux of cholesterol from astrocytes, is predominantly mediated by ATP‐binding cassette (ABC) transporters, particularly ABCA1 and ABCG1, by lipidating cholesterol onto lipid‐poor apolipoproteins, primarily APOE (Lewandowski et al. 2022). This process facilitates the removal of excess free cholesterol from astrocytes and prevents intracellular cholesterol accumulation.

#### 2.3.2 Cholesterol Redistribution

APOE serves as the principal cholesterol carrier in the brain and is synthesized in the astrocytes. APOE lipidation is driven primarily by ABCA1 and ABCG1, which load phospholipids and cholesterol onto nascent APOE particles. These APOE‐lipidated particles then travel among astrocytes, microglia, and neurons, supplying cholesterol for neuronal membrane maintenance and synaptic function (Husain et al. 2021).

#### 2.3.3 Cholesterol Influx

Neurons and glia take up cholesterol through members of the low‐density lipoprotein (LDL) receptor family, with LDL receptor‐related protein 1 (LRP1) being the most prominent (Lane‐Donovan et al. 2014).

### 2.4 Intracellular Cholesterol Transport

In addition to synthesis, metabolism, and intercellular transport, cholesterol homeostasis also depends on efficient intracellular trafficking between the plasma membrane, endoplasmic reticulum, endosomes, and lysosomes (Soccio and Breslow 2004; Meng et al. 2020). Following receptor‐mediated uptake of APOE‐containing lipoproteins, cholesterol must be mobilized through the endolysosomal system for redistribution, membrane repair, and feedback regulation of sterol‐sensitive pathways. Disruption of endolysosomal cholesterol trafficking may therefore uncouple cellular cholesterol content from sterol sensing, promoting lipid accumulation, defective recycling, and impaired membrane homeostasis (Meng et al. 2020).

## 3 Drivers of Cholesterol Dyshomeostasis: When the System “Breaks”

**FIGURE 4:**
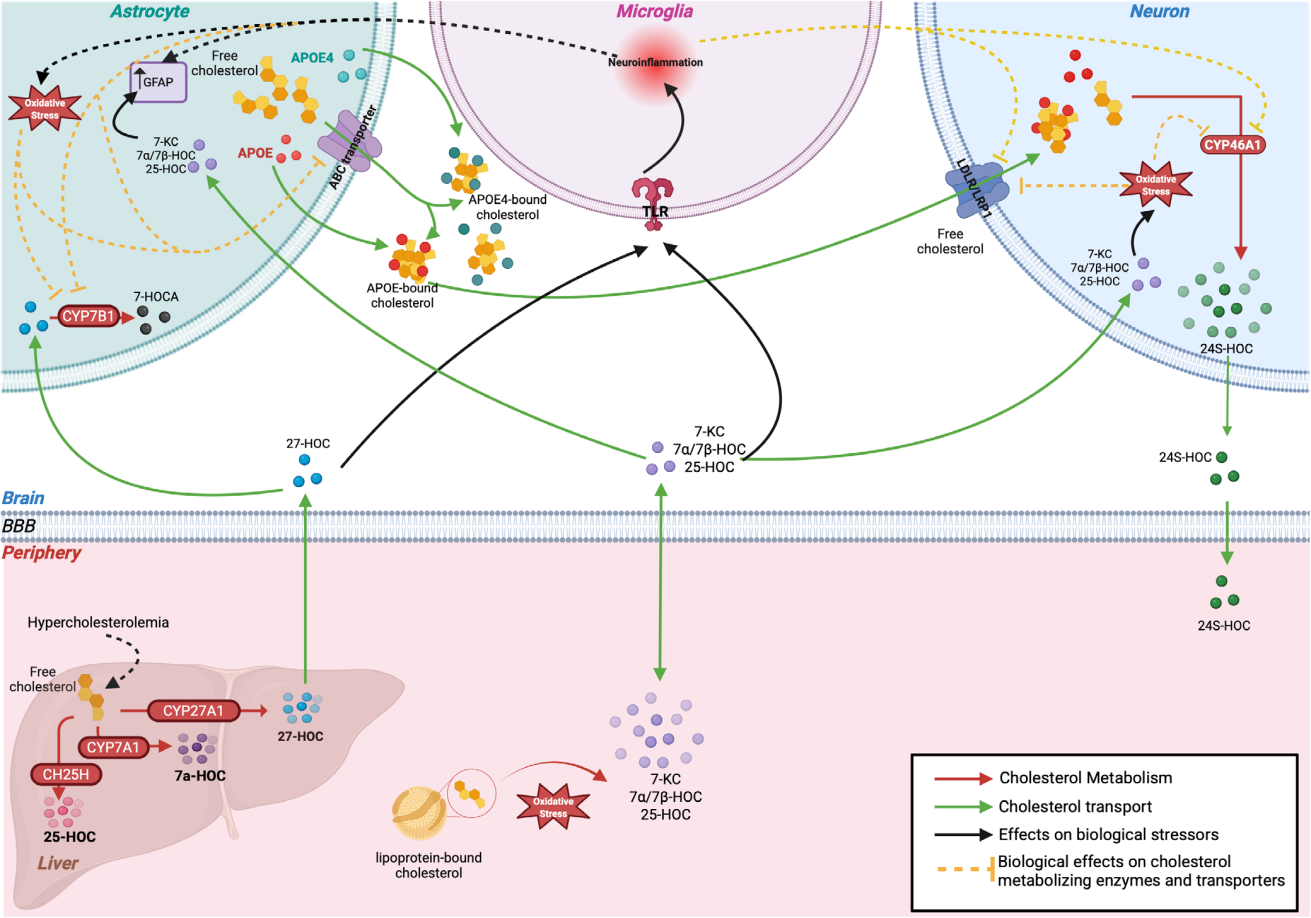
Overview of biological stressors on mechanisms regulating cholesterol homeostasis. Biological stressors perturb cholesterol homeostasis by disrupting synthesis, metabolism, and transport across astrocytes, neurons, and microglia. Peripheral oxysterols, including 27‐HOC, enter the brain and perturb sterol balance, while oxidative and inflammatory processes promote the formation of cytotoxic oxysterols (e.g., 7‐KC, 7α/7β‐HOC). These stressors impair APOE‐mediated lipid transport, disrupt CYP46A1‐dependent cholesterol turnover, and promote glial lipid accumulation and inflammatory activation. Of importance, In addition to its role in cholesterol elimination, 24S‐HOC also acts locally within the CNS to regulate lipid metabolism, inflammatory signaling, and LXR‐dependent transcriptional pathways in astrocytes and microglia. Collectively, these processes drive a transition from tightly regulated cholesterol homeostasis to a state of sterol imbalance, impaired clearance, and dysfunctional lipid trafficking, linking systemic and central stressors to AD pathophysiology.

Multiple converging stressors disrupt cholesterol homeostasis in the aging and AD brain. Hypercholesterolemia, neurodegeneration, oxidative stress, and inflammatory injury each perturb different components of synthesis, metabolism, and transport (Figure 4). Together, these processes shift the brain from a tightly regulated system to one characterized by sterol imbalance, impaired clearance, and dysfunctional cholesterol trafficking.

### 3.1 Aging

AD and related neurodegenerative disorders occur almost exclusively in the context of advancing biological age, suggesting that age‐related declines in cellular resilience and homeostatic regulation create a permissive environment for neurodegeneration. Aging therefore represents the most significant biological driver of cholesterol dyshomeostasis in the brain. Even in the absence of overt neurodegeneration, aging is associated with reduced neuronal cholesterol synthesis capacity, increased reliance on astrocyte‐derived cholesterol, and altered coordination of cholesterol turnover pathways involving CYP46A1‐mediated metabolism (Morgan et al. 2016; Allende and Natalí 2024). These changes do not necessarily reflect complete dysfunction, but rather a reduction in the flexibility and resilience of cholesterol regulatory systems, increasing susceptibility to additional stressors such as inflammation, oxidative stress, and metabolic dysregulation. At the level of cholesterol synthesis, aging is associated with a progressive decline in neuronal cholesterol production (Thelen et al. 2006; Boisvert et al. 2018), accompanied by an increased dependence on astrocyte‐derived cholesterol. This reflects reduced expression and activity of key cholesterol synthesis enzymes in neurons, together with altered sterol sensing mechanisms that may limit the ability of neurons to dynamically adjust cholesterol synthesis in response to metabolic demand. Although astrocytes may partially compensate for this decline, age‐related changes in astrocytic function can impair the efficiency and adaptability of this support, contributing to regional and cell‐type–specific imbalances in cholesterol availability (Boisvert et al. 2018).

Aging also alters cholesterol metabolism and turnover. While CYP46A1 remains the primary pathway for cholesterol elimination from the brain, age‐related effects are more consistently observed at the level of cholesterol flux rather than intrinsic enzymatic efficiency. This is reflected in reported changes in circulating and cerebrospinal fluid concentrations of 24S‐HOC across aging and neurodegenerative contexts, suggesting altered coordination between cholesterol production and clearance (Lütjohann and Breuer 1996; Wang and Wang 2016). In parallel, aging is associated with increased vulnerability to the accumulation of oxysterols generated through oxidative and enzymatic pathways, including 7‐KC and 25‐HOC, which promote cellular stress, inflammation, and further disruption of sterol homeostasis (Anderson and Campo 2020; Zarrouk and Vejux 2014).

Intercellular cholesterol transport pathways are also sensitive to aging‐related changes in system efficiency. Cholesterol efflux from astrocytes is mediated primarily by ATP‐binding cassette transporters, including ABCA1 and ABCG1, which facilitate the transfer of lipids to APOE‐containing lipoprotein particles (Berrougui et al. 2007). Proper lipidation of APOE is critical for its function as a cholesterol carrier, and disruption of ABCA1‐dependent lipid efflux markedly impairs APOE lipidation and secretion, leading to altered cholesterol distribution within the brain (Berrougui et al. 2007; de Chaves and Narayanaswami 2008). In this context, neuronal cholesterol balance reflects the integration of APOE‐mediated cholesterol delivery and CYP46A1‐mediated elimination, linking intercellular transport to intracellular cholesterol turnover. While these transport mechanisms remain operational during aging, evidence suggests that their efficiency and adaptability may be reduced, particularly under conditions of metabolic or oxidative stress, which are processes that are exacerbated with aging (Laguna‐Maldonado et al. 2025; Liguori and Russo 2018). Notably, impairments in ABCA1‐ and ABCG1‐mediated cholesterol efflux and APOE lipidation are well documented in neurodegenerative conditions such as AD (Abuznait and Kaddoumi 2012; Pereira et al. 2018), where reduced cholesterol transport capacity contributes to neuronal vulnerability. Thus, aging may not directly abolish these pathways but instead creates a state in which intercellular cholesterol transport becomes increasingly susceptible to dysfunction. This progressive reduction in transport efficiency may become particularly consequential when combined with other age‐related stressors, including inflammation and oxidative damage, which further destabilize cholesterol homeostasis.

These age‐related changes create a metabolic environment in which cholesterol homeostasis becomes increasingly dependent on intact cellular stress response pathways, rendering the system particularly vulnerable to oxidative and inflammatory insults.

### 3.2 Sex

Sex differences are well recognized in AD, with females exhibiting a greater lifetime risk and often more rapid cognitive decline and pathological progression compared with males, differences that are not fully explained by increased longevity (Mielke 2018). These observations support the importance of sex‐dependent biological mechanisms in modulating lipid metabolism, inflammatory signaling, and vulnerability to cholesterol dyshomeostasis in aging and AD. Sex is therefore a critical biological variable influencing cholesterol metabolism, lipid transport, and neuroinflammatory responses in the brain. Estrogen signaling enhances ABCA1‐mediated cholesterol efflux and promotes APOE lipidation, thereby supporting efficient intercellular lipid transport and neuronal repair (Srivastava et al. 1997; Srivastava 2002). In addition, estrogen interacts with LXR signaling pathways, linking cholesterol transport to anti‐inflammatory transcriptional programs and coordinating lipid handling with immune regulation (Fan and Shimizu 2013). Beyond estrogen, androgen signaling (Cardoso and Figueira 2022; Pace and Werz 2020) and sex chromosome–linked mechanisms (Link and Chen 2015; Tabassum et al. 2023; Bobotis and Khakpour 2025) may also contribute to sex differences in lipid metabolism and inflammatory responses, although these pathways remain less well characterized in the context of brain cholesterol homeostasis.

Microglia exhibit well‐documented sex‐specific differences in lipid metabolism and activation states, with estrogen generally promoting a more homeostatic lipid‐handling phenotype, while reduced estrogen levels are associated with increased lipid droplet accumulation, impaired lipid efflux, and pro‐inflammatory responses (Cleland and Potter 2024). These differences influence how microglia respond to cholesterol and oxysterol accumulation, positioning sex as a key modifier of lipid‐driven neuroinflammatory signaling in aging and AD.

In addition to effects on lipid transport, sex hormones may also influence cholesterol synthesis and turnover pathways through modulation of sterol‐sensitive transcriptional regulators, including SREBP and LXR signaling (Fernández‐Suárez and Daimiel 2021; Krycer and Brown 2011). These effects suggest that sex‐dependent differences extend across multiple components of cholesterol homeostasis, rather than being restricted to a single pathway. Importantly, age‐related declines in estrogen, such as those occurring during menopause, are likely to exacerbate age‐related impairments in cholesterol synthesis, transport, and inflammatory regulation, thereby amplifying vulnerability to cholesterol dyshomeostasis and AD. Collectively, these mechanisms may contribute to observed sex differences in disease risk and progression, particularly among APOE4 carriers.

### 3.3 Peripheral Hypercholesterolemia

Cholesterol accumulation due to peripheral hypercholesterolemia is an extrinsic driver of cholesterol sterol dysregulation by overwhelming homeostasis feedback systems that normally couple neuronal cholesterol demand to local synthesis.

#### 3.3.1 Impact on Cholesterol Synthesis

Elevated cholesterol and its metabolites accumulate in the ER and plasma membrane and activate INSIG‐mediated retention of the SCAP‐SREBP2 complex, and promote degradation of HMGCR and SQLE (Radhakrishnan et al. 2008). Consequently, cholesterol synthesis declines despite elevated neuronal demands during neurodegeneration‐induced sterol accumulation.

#### 3.3.2 Impact on Cholesterol Metabolism

Elevated peripheral cholesterol increases the production of 27‐HOC (Heverin and Meaney 2005), and its flux into the brain, disrupting local sterol composition and impairing CYP27A1 and CYP46A1 activity (Li et al. 2007; Milagre and Olin 2012). Experimental models demonstrate that excess 27‐HOC disrupts the balance between cholesterol synthesis and turnover, contributing to a metabolic state characterized by brain sterol accumulation and reduced cholesterol clearance (Wu and Zhai 2022).

#### 3.3.3 Impact on Cholesterol Transport

Increased sterol burden may overwhelm membrane‐remodeling processes (Subczynski et al. 2017) and ATP‐dependent lipid transporters that maintain proper cholesterol distribution (Oram and Vaughan 2006). Changes in membrane sterol composition can alter the activity of flippases, floppases, and scramblases, enzymes responsible for maintaining phospholipid and cholesterol asymmetry across the bilayer (Sakuragi and Nagata 2023). Furthermore, cholesterol accumulation in the plasma membrane may reduce APOE–lipoprotein particle loading efficiency and decrease ABCA1/ABCG1‐mediated cholesterol efflux (Yang et al. 2023), thereby compromising astrocyte‐to‐neuron cholesterol transport.

### 3.4 Neurodegeneration

Neuronal injury and death are themselves potent disruptors of cholesterol homeostasis. As neurons contain high levels of unesterified cholesterol integrated into axonal and synaptic membranes, neurodegeneration releases large amounts of cholesterol and sterol precursors into the local microenvironment (Hughes et al. 2013). This passive sterol efflux adds to the burden created by hypercholesterolemia and accelerates failure of synthesis and metabolic pathways.

#### 3.4.1 Impacts on Cholesterol Synthesis

Neurodegeneration shifts the local sterol environment and reinforces feedback inhibition of SREBP2 processing. As degenerating neurons release cholesterol and sterol intermediates, the ER and plasma membrane become sterol‐enriched, suppressing HMGCR and SQLE and impairing the ability of both neurons and glia to maintain cholesterol synthesis. In astrocytes, this disruption further compromises the cholesterol export required to support vulnerable neurons.

#### 3.4.2 Impacts on Cholesterol Metabolism

With neurodegeneration, the production of CYP46A1 decreases (Boussicault and Alves 2016). As such, the brain's ability to eliminate cholesterol is reduced, which is reflected in the changes in 24S‐HOC observed in neurodegenerative diseases (Lütjohann and Papassotiropoulos 2000; Papassotiropoulos and Lütjohann 2002; Gamba and Giannelli 2021).

#### 3.4.3 Impacts on Cholesterol Transport

As neurodegeneration increases the local sterol pool, this disrupts cholesterol transport from astrocytes and microglia. APOE‐containing lipoproteins become smaller and less lipid‐rich, compromising delivery of cholesterol to surviving neurons and thereby impairing synaptic maintenance, even in the context of already constrained neuronal synthetic capacity (Yang et al. 2023).

### 3.5 Oxidative Stress and Inflammation

Oxidative stress and inflammation are biological processes that are implicated in aging and neurodegenerative diseases such as AD, which can have significant impacts on cholesterol dyshomeostasis.

#### 3.5.1 Impacts on Cholesterol Synthesis

Oxidative stress and inflammation enhance HMGCR and SQLE degradation through enhanced ER‐associated protein degradation (Liu and Zhang 2024; Cao and Kaufman 2014; Cao et al. 2016), while also inhibiting DHCR24 in astrocytes and DHCR7 in neurons (Bai and Mai 2022). Thus, oxidative and inflammatory injury simultaneously restrict both presqualene and post‐squalene cholesterol synthesis while increasing cellular vulnerability through toxic sterol buildup.

#### 3.5.2 Impacts of Cholesterol Metabolism

Oxidative stress and inflammation disrupt cholesterol metabolism through both enzymatic impairment and increased non‐enzymatic oxysterol formation. Reactive oxygen species, lipid peroxides, free radicals, and metal ions drive the generation of oxysterols from auto‐oxidative pathways which include 7‐ketocholesterol (7‐KC), 7α/7β‐hydroxycholesterol (7α/7β‐HOC), and 25‐hydroxycholesterol (25‐HOC) (Zerbinati and Iuliano 2017). These species are cytotoxic, pro‐inflammatory, and accumulate in both the periphery and brain (Zerbinati and Iuliano 2017; Huang et al. 2014), providing a mechanistic link between oxidative stress and inflammation, cholesterol dysregulation, and neurodegeneration. Oxidative stress and inflammation also impact the function of CYP46A1 and CYP27A1, key cholesterol metabolizing enzymes in the brain and periphery, respectively. This further impairs cholesterol turnover and contributes to sterol accumulation.

#### 3.5.3 Impacts on Cholesterol Transport

Oxidative stress oxidizes membrane cholesterol and phospholipids (Itri et al. 2014; Schumann‐Gillett and O'Mara 2019), impairing fluidity and function of lipid transporters. Oxidized membranes destabilize ABCA1 and ABCG1 activity, diminishing APOE lipidation and compromising the formation of cholesterol‐rich lipoproteins, while also destabilizing LRP1‐mediated uptake (Oram and Heinecke 2005; Owen and Sultana 2010; Laguna‐Maldonado et al. 2025). Inflammation further amplifies these deficits as cytokines such as tumor necrosis factor and interleukin 1‐beta downregulate ABCA1 expression (Chen et al. 2007), subsequently impairing astrocytic cholesterol efflux. Oxysterols such as 7‐KC also impair astrocytic cholesterol export and can induce microglial lipid droplet accumulation, further reducing lipid transport efficiency (Gaus et al. 2001). Collectively, oxidative and inflammatory damage disrupts astrocyte‐to‐neuron lipid trafficking and impairs membrane repair processes required for synaptic resilience.

## 4 Consequences for AD

Cholesterol homeostasis is essential for maintaining neuronal function, synaptic plasticity, membrane integrity, and lipid signaling in the healthy brain. Biological stressors disrupt this balance by impairing cholesterol synthesis, metabolism, and transport. This results in sterol imbalance which initiates and amplifies multiple pathological processes that drive AD. The following sections describe how these disruptions translate into downstream molecular, cellular, and systems‐level consequences relevant to AD.

### 4.1 Cholesterol Synthesis

Cholesterol synthesis becomes increasingly dysregulated in AD, and this loss of coordinated regulation contributes to disease vulnerability. Under normal conditions, elevated intracellular cholesterol suppresses SREBP2 activation and reduces expression of HMGCR, SQLE, and DHCR24. However, in AD, multiple stressors, such as an increased sterol environment and the presence of elevated oxidative stress and inflammation, disrupt this regulatory loop in cell‐type–specific ways.

Neuronal injury has been associated with reduced SREBP2 (Kim and Ong 2009) and DHCR24 expression (Bai and Mai 2022). DHCR24 has also shown age‐ and region‐specific downregulation in the hippocampus, an AD‐vulnerable brain region (Iivonen and Hiltunen 2002). Loss of DHCR24 limits post‐squalene conversion to cholesterol, promotes accumulation of toxic intermediates such as desmosterol and 7‐DHC, and reduces the cholesterol needed for membrane repair and synaptic maintenance (Huang and Zhang 2023). Because DHCR24 also exerts anti‐oxidant and anti‐apoptotic functions, its decline increases susceptibility to tau pathology and oxidative injury.

In contrast, astrocytes in plaque‐rich regions have exhibited an upregulation of HMGCR (Azizidoost et al. 2022), reflecting local stress and disrupted homeostatic regulation. This localized increase may not compensate for neuronal deficits; instead, it may indicate that the normal sterol‐sensing mechanisms governing synthesis are decoupled from cellular needs, resulting in toxic cholesterol accumulation.

Genetic evidence reinforces the relevance of the synthesis pathway to AD. Polymorphisms in HMGCR, such as the rs3846662 polymorphism, have been associated with altered HMGCR splicing, elevated plasma cholesterol, and increased AD risk (Leduc and de Beaumont 2015). This suggests that the vulnerability of the synthetic machinery, rather than a uniform direction of change, modulates susceptibility to neurodegeneration.

### 4.2 Cholesterol Metabolism

Alterations in cholesterol metabolism, particularly involving CYP46A1 and CYP27A1, have profound implications for AD. As the primary route of cholesterol turnover in the brain, CYP46A1‐derived 24S‐HOC is a direct marker of brain cholesterol turnover (Lütjohann and Papassotiropoulos 2000). Changes in 24S‐HOC have been observed across the AD continuum. Specifically, 24S‐HOC exhibits a biphasic profile where concentrations increase during the early stages of cognitive impairment reflecting compensatory upregulation (Papassotiropoulos and Lütjohann 2002; Gamba and Giannelli 2021; Lütjohann and Papassotiropoulos 2000), and then decline with increasing AD severity as neuronal loss and reduced CYP46A1 expression limit cholesterol turnover (Gamba and Giannelli 2021). Experimental studies support these associations as CYP46A1 inhibition in mice has been associated with increased Ab accumulation and cognitive decline (Djelti and Braudeau 2015), while oxidative stress upregulates CYP46A1 transcription (Ohyama and Meaney 2006), which may reflect a compensatory response to increased central stress. In contrast, in vitro studies have demonstrated that proinflammatory cytokines such as TNF‐alpha, suppress CYP46A1 expression (Russell et al. 2009). Additional studies are needed to determine if these findings reflect distinct cellular responses to different biological stressors, ultimately impairing cholesterol metabolism during sustained states of oxidative or inflammatory stress.

Genetic studies further implicate *CYP46A1* variation in modifying AD risk. Specifically, rs7157609 (Kölsch and Lütjohann 2009), rs754203 (Johansson and Katzov 2004), rs4900442 (Jia and Liu 2016) have been associated with increased 24S‐HOC and increased AD risk, suggesting that genetic modulation of CYP46A1 impacts both cholesterol turnover and AD risk. Together, these data position CYP46A1 as a critical metabolic node linking cholesterol regulation to AD progression.

In parallel, 27‐HOC, a CYP27A1‐derived oxysterol, also contributes to AD pathophysiology. Elevated peripheral cholesterol increases 27‐HOC flux into the brain, where it can disrupt central cholesterol homeostasis, promote Abeta accumulation (Zhang and Xi 2019) and inflammation pathways through activation of the NLRP3 (NOD‐, LRR‐ and pyrin domain containing protein 3) inflammasome and subsequent expression of proinflammatory inflammatory mediators such as IL‐1β, TNF‐α, and RAGE (Zhang and Xi 2019; Son and Yeo 2023; Loera‐Valencia and Ismail 2021). Unlike 24S‐HOC, which decreases with dementia severity due to neuronal loss, 27‐HOC levels increase with advancing disease, reflecting sustained peripheral input and impaired CNS clearance due to reduced CYP7B1 expression in the AD brain (Yau and Rasmuson 2003). Experimental models support these findings as CYP27A1 overexpression and consequently chronic 27‐HOC exposure impair neuronal morphology, synaptic density, and cognition (Wu and Zhai 2022; Alanko and Gaminde‐Blasco 2023). Furthermore, CYP7B1 knockout mice accumulate 27‐HOC (Goikolea and Latorre‐Leal 2023), suggesting that toxicity may depend on local enzymatic context and oxysterol balance.

Finally, elevated concentrations of oxysterols generated through oxidative stress, which include 7‐KC, 7β‐HOC, and 25‐HOC, have been detected in the brains and plasma of individuals with AD and MCI (Vejux and Ghzaiel 2025; Choi and Kim 2025). These oxysterols contribute to neuronal dysfunction, mitochondrial injury, and microglial activation, further amplifying oxidative and inflammatory cascades that accelerate neurodegeneration. 7‐KC and 7β‐HOC, in particular, disrupt membrane integrity and induce apoptosis in neurons and astrocytes (Anderson and Campo 2020; Kupferberg and Teller 1989), while 25‐HOC promotes inflammatory cytokine production through TLR–NFκB and NLRP3 inflammasome activation (Choi and Kim 2025). Experimental studies demonstrate that exposure to 7‐KC or 25‐HC increases Aβ production, tau phosphorylation, and lipid peroxidation, supporting a mechanistic link between oxidative cholesterol metabolism and AD pathology (Phan et al. 2018; Lee and Kim 2024; Toral‐Rios and Long 2024; Ha and Kwon 2024). While studies have not extensively focused on 7α‐HOC, clinical studies have reported that individuals deficient in CYP7A1 have elevated total and LDL cholesterol, and cholesterol in the liver (Pullinger and Eng 2002); characteristics of hyperlipidemia, which is an established risk factor of dementia (Livingston and Huntley 2024). Thus, dysregulated cholesterol metabolism, whether through impaired enzymatic clearance or uncontrolled oxidative pathways, acts as a significant contributor to neurodegeneration.

### 4.3 Cholesterol Transport

Disruptions in cholesterol transport represent a final, critical link between cholesterol dyshomeostasis and AD pathogenesis. Impaired ABCA1/ABCG1‐mediated efflux, poor APOE lipidation, and impaired LRP1‐mediated uptake together create a lipid‐locked state that restricts cholesterol availability to neurons while promoting glial lipid accumulation and inflammation. These changes favor amyloidogenic APP processing by impacting the composition of the lipid bilayer, impairing synaptic repair, and potentiating neuroinflammatory cascades (Kim and Rahmanto 2007; Yvan‐Charvet et al. 2010; Bossaerts et al. 2022), further propagating AD pathology. Defects in endolysosomal cholesterol trafficking may further exacerbate these transport‐related abnormalities (Meng et al. 2020).

*APOE4* remains the strongest genetic determinant of late‐onset AD, and its role in lipid biology is central to this risk (Di Battista et al. 2016). *APOE4* is the least efficiently lipidated, producing smaller, unstable lipoprotein particles that impair cholesterol delivery to neurons and reduce support for synaptic repair. This deficit becomes especially consequential under conditions of oxidative or metabolic stress, when the demand for lipid trafficking increases. Clusterin (apolipoprotein J, *CLU*), another top AD‐risk gene (Foster et al. 2019), contributes to lipoprotein remodeling, membrane stabilization, proteotoxic stress responses, and extracellular Aβ chaperoning. Although *CLU* is not responsible for bulk cholesterol transport, its roles in lipid handling intersect directly with APOE‐dependent pathways. Together, the genetic architecture of *APOE4* and *CLU* underscores a core principle emerging across AD biology: that vulnerability to AD arises from reduced capacity to maintain cholesterol homeostasis under stress, linking lipid dysregulation to impaired synaptic maintenance, heightened inflammation, and amyloidogenic processing. These transport‐related vulnerabilities may also be modified by sex, particularly in APOE4 carriers, in whom cholesterol trafficking deficits and AD risk appear to be more pronounced in females than in males (Ungar et al. 2014).

Intracellularly, cholesterol homeostasis is critically dependent on trafficking through the endolysosomal system. Following APOE‐mediated uptake, cholesterol is routed through endosomes and lysosomes before redistribution to the endoplasmic reticulum and plasma membrane. In AD, disruption of this pathway leads to sequestration of cholesterol within endolysosomal compartments, effectively uncoupling cellular cholesterol content from sterol sensing and feedback regulation. This compartmental imbalance alters the localization and trafficking of APP, BACE1, and γ‐secretase, which reside and traffic through endosomal membranes, thereby promoting amyloidogenic processing. In parallel, impaired endolysosomal cholesterol trafficking disrupts membrane recycling and receptor turnover, further exacerbating synaptic dysfunction and neurodegenerative signaling (Meng et al. 2020).

### 4.4 Cell‐Type–Specific Manifestations of Cholesterol Dyshomeostasis

**FIGURE 5:**
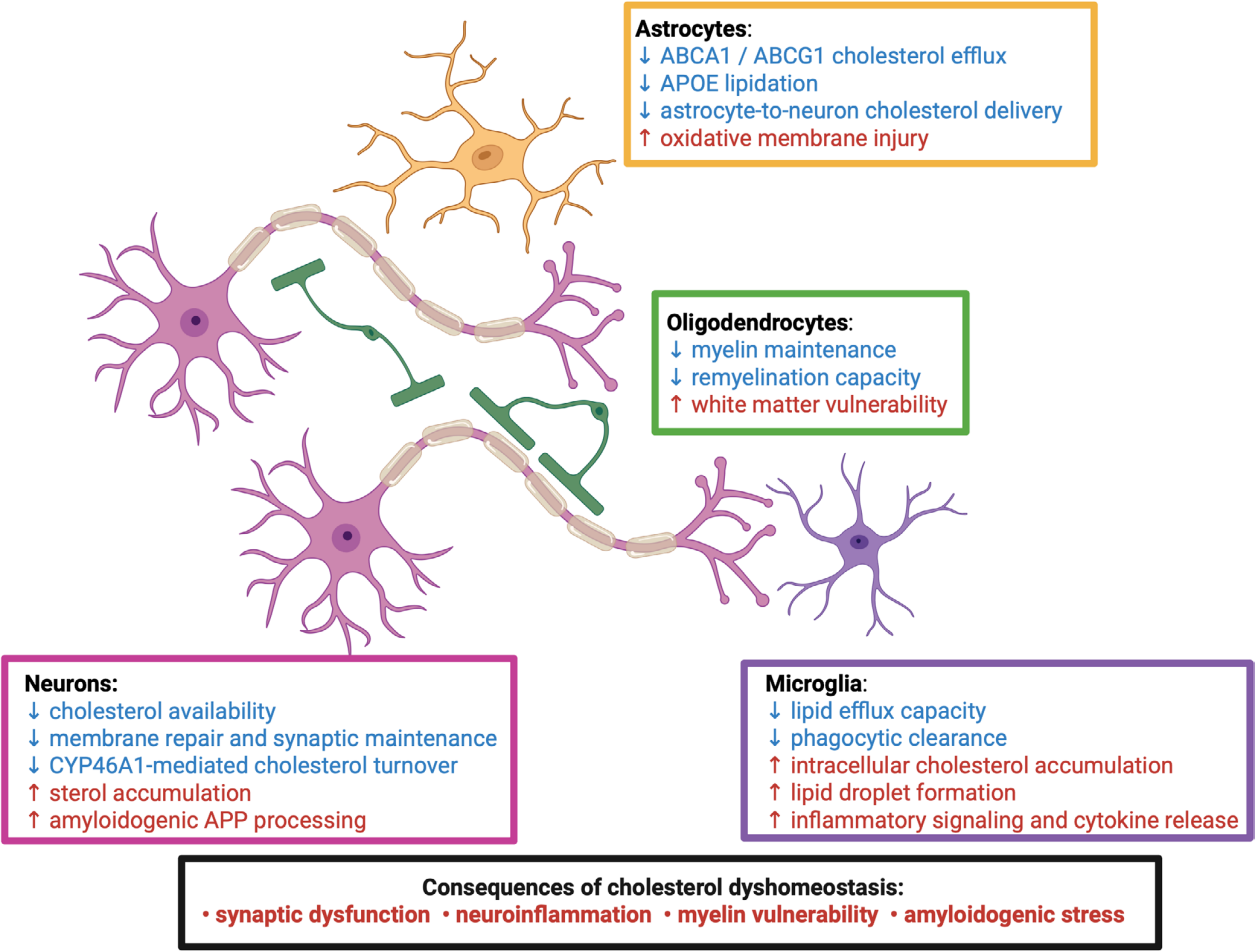
Cell‐type–specific consequences of cholesterol dyshomeostasis in Alzheimer's disease. Neurons, astrocytes, microglia, and oligodendrocytes exhibit distinct alterations in cholesterol metabolism, transport, and lipid handling in AD. These cell‐specific disruptions collectively contribute to synaptic dysfunction, neuroinflammation, myelin vulnerability, and amyloidogenic stress.

Although cholesterol dyshomeostasis can be described through disruptions in its synthesis, metabolism, and transport, its pathological consequences in AD are strongly cell‐type specific. The major brain cell types, neurons, astrocytes, microglia, and oligodendrocytes differ in their cholesterol synthetic capacity, dependence on lipid transport, and vulnerability to sterol imbalance, resulting in distinct but interconnected contributions to disease progression (Figure 5).

#### 4.4.1 Neurons

Neurons rely increasingly on exogenous cholesterol support with aging and are particularly vulnerable to impaired cholesterol transport and turnover (Genaro‐Mattos et al. 2019). Reduced neuronal cholesterol availability compromises membrane repair (Berghoff and Spieth 2021), synaptic integrity (Shaheen et al. 2023), vesicle trafficking (Wasser et al. 2007), and APP processing (Beel et al. 2010), while impaired CYP46A1‐mediated turnover promotes sterol accumulation and amyloidogenic stress (Djelti and Braudeau 2015).

#### 4.4.2 Astrocytes

Astrocytes are the principal source of *de novo* cholesterol synthesis in the adult brain and play a central role in APOE lipidation and cholesterol efflux. Under AD‐related stress, impaired ABCA1/ABCG1 function (Marchi and Adorni 2019), altered APOE lipidation (Lanfranco et al. 2020), and oxidative membrane injury (Baumart and Rodrigues 2026) reduce the efficiency of astrocyte‐to‐neuron cholesterol delivery, thereby weakening neuronal support and promoting extracellular lipid disequilibrium.

#### 4.4.3 Microglia

Microglia integrate lipid sensing with innate immune activation (Chausse et al. 2021). Excess cholesterol and oxysterol exposure promote inflammatory signaling, while impaired lipid efflux favors intracellular lipid accumulation and formation of lipid droplets (Haney and Pálovics 2024). These lipid droplet–associated microglial states are increasingly recognized as dysfunctional phenotypes linked to defective phagocytosis, sustained cytokine release, and propagation of neurodegenerative injury (Nanjundaiah et al. 2021).

#### 4.4.4 Oligodendrocytes

Oligodendrocytes contain and require large quantities of cholesterol to sustain myelin production and maintenance (Kuhn et al. 2019). Because most brain cholesterol resides in myelin, disruption of cholesterol homeostasis may impair myelin integrity, remyelination capacity, and white matter resilience. These effects are particularly relevant in AD, where white matter injury and myelin vulnerability may interact with neurovascular dysfunction and neurodegeneration (Chen and Yi 2013; Hamanaka et al. 2018).

The impact of cholesterol dyshomeostasis across different cell‐types indicates that it is not a uniform process across the CNS, but rather a multicellular failure of lipid coordination that differentially affects neuronal maintenance, glial reactivity, and myelin stability.

### 4.5 Other AD‐Relevant Consequences

Cholesterol homeostasis maintains a tightly regulated balance between synthesis, metabolism, and transport to support membrane organization, synaptic transmission, vesicle recycling, mitochondrial stability, and glial–neuronal lipid coupling. Stressors that perturb this balance, such as sterol accumulation due to hypercholesterolemia and/or neurodegeneration, oxidative stress, and inflammation, shift the brain toward a state of cholesterol dyshomeostasis. These disturbances reshape membrane architecture, amplify innate immune activation, and impair cerebrovascular resilience, creating a biochemical environment that facilitates amyloidogenic processing, tau pathology, and neurodegeneration.

#### 4.5.1 Lipid Bilayer Integrity and Amyloidogenic Processing

Neuronal and glial membranes are composed of a complex mixture of cholesterol and phospholipids containing polyunsaturated fatty acids (PUFAs) that determine bilayer fluidity and function. The ratio of cholesterol to PUFAs is critical as excess cholesterol stiffens the membrane, reducing flexibility and altering the organization of lipid raft domains, while PUFA enrichment increases fluidity but can also predispose membranes to oxidative damage (Pamplona and Portero‐Otín 1998). Disruption of cholesterol homeostasis, such as reduced CYP46A1 activity and/or impaired cholesterol transport, can increase membrane cholesterol content and impair this dynamic balance, compromising receptor trafficking and vesicular signaling.

Phospholipid asymmetry is maintained by flippases, floppases (including ABCA1/ABCG1), and scramblases, which regulate cholesterol and PUFA distribution between the inner and outer membrane leaflets. Cholesterol accumulation or oxidative stress can impair these transporters (Sakuragi and Nagata 2023; Menon et al. 2024), limiting lipid remodeling and consequently reducing the efficiency of glial APOE lipidation (Yang et al. 2023; Rebeck 2017). This would occur at the astrocytic plasma membrane and within endosomal compartments, where nascent APOE particles acquire cholesterol and phospholipids prior to selection. In addition, PUFAs also serve as precursors for a broad array of lipid mediators. Cytochrome P450 (CYP450) epoxygenases (CYP2C and CYP2J families) convert PUFAs into epoxides with vasoprotective and anti‐inflammatory properties.

**FIGURE 6:**
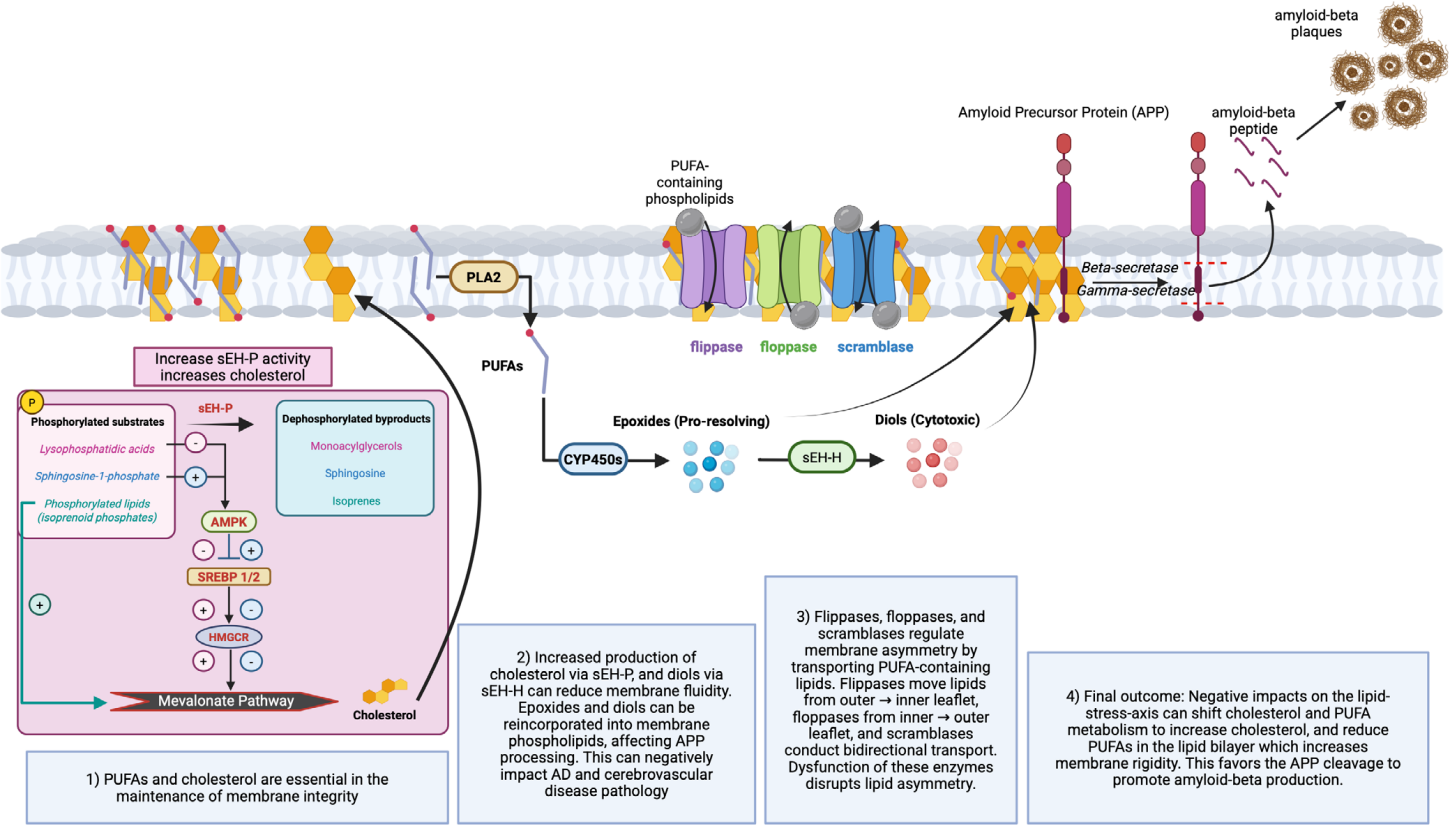
Overview of the role of sEH in cholesterol synthesis, membrane integrity, and processing of APP. sEH integrates PUFA metabolism with cholesterol‐dependent membrane regulation. CYP450 epoxygenases convert PUFAs into bioactive epoxides with anti‐inflammatory and vasoprotective properties, which are subsequently metabolized by the hydrolase domain of sEH into less active or cytotoxic diols. This shift promotes oxidative stress and inflammation and alters membrane lipid composition. Changes in cholesterol–PUFA balance affect membrane fluidity, lipid raft organization, and the localization of APP‐processing enzymes, including β‐secretase and γ‐secretase, thereby favoring amyloidogenic processing. In parallel, the phosphatase domain of sEH is implicated in intracellular lipid signaling pathways that regulate sterol‐sensitive mechanisms, including SREBP2‐dependent cholesterol synthesis. Together, sEH represents a key point of convergence between lipid metabolism, membrane integrity, and AD‐related pathophysiology. While sEH is the primary enzyme mediating epoxide hydrolysis in this context, microsomal epoxide hydrolase (mEH) may also contribute to epoxide metabolism within the endoplasmic reticulum. In addition, the subcellular localization of sEH, which can associate with the endoplasmic reticulum and cytosol, may influence its access to lipid substrates and its impact on membrane lipid dynamics.

Through its hydrolase domain, soluble epoxide hydrolase (sEH), the predominant cytosolic epoxide hydrolase in the CNS, converts these epoxides into less active or cytotoxic diols, tipping the balance toward oxidative stress and inflammation (Newman et al. 2003). Through its phosphatase domain, sEH is implicated in regulating intracellular lipid‐related signaling pathways that influence cholesterol homeostasis, including modulation of sterol‐sensitive regulatory mechanisms such as SREBP2 activation and downstream expression of cholesterol synthesis enzymes (EnayetAllah and Luria 2008; Domingues et al. 2019; Messaoudi and Varennes 2025) (Figure 6). Collectively, sEH is a key point of convergence between cholesterol and PUFA regulation; a coordinated axis of lipid stress in AD. Accumulation of sEH‐derived diols may further exacerbate cellular lipid stress by promoting inflammatory activation and impairing lipid handling pathways, thereby contributing to a lipid stress environment that is associated with lipid accumulation and lipid droplet formation in glial cells (Olzmann and Carvalho 2019).

Within this altered lipid environment, APP is embedded in cholesterol‐ and PUFA‐rich bilayers. Shifts in membrane composition alter the localization of BACE1 and γ‐secretase, driving APP processing toward the amyloidogenic pathway, increasing Aβ generation (Zhang and Song 2013), and tau pathology (Chen and Fan 2023). Dysregulation of cholesterol‐PUFA balance and sEH activity therefore represents a mechanistic link between altered membrane lipid remodeling due to cholesterol dyshomeostasis and AD pathogenesis. These alterations in membrane lipid composition and trafficking also extend to glial cells, where impaired lipid handling and chronic inflammatory signaling contribute to pathological lipid storage phenotypes (Miller et al. 2020).

In addition to cholesterol‐phospholipid interactions, membrane organization in the CNS is also critically governed by the sphingolipid–cholesterol axis. Cholesterol and sphingomyelin co‐accumulate within lipid raft domains, where they regulate membrane order, receptor clustering, and trafficking of signaling complexes (Fantini and Barrantes 2009; Fantini and Yahi 2010). Disruption of this axis, through altered sphingomyelin metabolism or increased ceramide generation, shifts membrane properties toward a more rigid and stress‐responsive state (Goñi and Alonso 2009). Ceramide enrichment, in particular, promotes stabilization of amyloidogenic processing machinery by facilitating the localization of BACE1 and γ‐secretase within ordered membrane domains, thereby enhancing Aβ production (Jazvinšćak Jembrek et al. 2015). In parallel, sphingolipid–cholesterol imbalance may impair membrane curvature, vesicular trafficking, and endosomal sorting, further linking lipid dysregulation to APP processing and neuronal vulnerability (Fantini and Yahi 2010; Brown and London 2000; Kim et al. 2026). Thus, cholesterol dyshomeostasis should be interpreted within a broader membrane lipid framework, in which sphingolipid interactions play a central role in shaping AD‐relevant membrane dysfunction.

#### 4.5.2 Neuroinflammation and Glial Activation

Cholesterol dysregulation and its metabolites are potent regulators of neuroinflammation. Oxysterols such as 27‐HOC and auto‐oxidized oxysterols (e.g., 7‐KC, 7β‐HOC) activate Toll‐like receptors (TLR) 2/4 and NLRP3 inflammasome signaling in microglia, leading to IL‐1β and IL‐18 cytokine release that propagate inflammatory signalling (Huang et al. 2014; Gargiulo and Gamba 2015; Erridge et al. 2007).

These oxysterols also act as endogenous ligands for LXRs, nuclear receptors that regulate cholesterol efflux (ABCA1/ABCG1) and suppress inflammatory gene expression (Fu and Menke 2001; Janowski and Grogan 1999). However, persistent oxysterol exposure in AD may lead to LXR desensitization, reduced ABCA1/ABCG1 expression, and impaired resolution of inflammation.

Microglial activation also stimulates the release of pro‐inflammatory cytokines and reactive oxygen species, which in turn activate astrocytes, resulting in astrogliosis (Zhang et al. 2010). Elevated GFAP expression has been observed in response to 25‐HOC and 27‐HOC in preclinical models (Romero et al. 2024; Hao and Wang 2023), supporting a direct link between oxysterol burden and glial reactivity.

An emerging feature of cholesterol‐related glial dysfunction in AD is the accumulation of intracellular lipid droplets, particularly in microglia and possibly astrocytes (Li and Zhao 2025). Recent evidence supports that lipid droplet–associated microglial states are linked to APOE4‐driven lipid remodeling and AD genetic risk pathways, reinforcing their role as a central feature of disease‐associated microglial dysfunction (Kozlova and Zhang 2025). Therefore, lipid droplet formation may be a downstream manifestation of unresolved lipid stress, in which excess cholesterol, fatty acids, and sEH‐derived diols are sequestered into neutral lipid stores under conditions of impaired lipid clearance (Wang and Zhao 2021; van der Vliet and de Vries 2026). Neutral lipids, including triglycerides and cholesteryl esters, are typically present at low levels in the brain, but their accumulation within lipid droplets reflects a shift toward pathological lipid storage under conditions of impaired lipid clearance. Consistent with this, recent lipidomic studies demonstrate increased triglyceride accumulation and lipid droplet formation in AD, but not in individuals with resilient pathology, suggesting that dysregulated lipid storage reflects a maladaptive response rather than a universal feature of amyloid burden (van der Vliet and de Vries 2026). Lipid droplet–associated microglia have been linked to defective phagocytosis, chronic inflammatory activation, and reduced metabolic fitness, suggesting that lipid droplet accumulation represents a maladaptive response that amplifies plaque‐associated pathology and neurodegeneration (Marschallinger and Iram 2020).

Triggering Receptor Expressed on Myeloid Cells 2 (TREM2), a microglial lipid‐sensing receptor, further integrates cholesterol handling with immune activation. TREM2 promotes cholesterol efflux through signaling the upregulation of ABCA1/ABCG1 (Wang et al. 2025). Reduced TREM2 function, which may occur due to increased inflammation (Okuzono and Sakuma 2021), impairs lipid uptake and clearance of apoptotic debris, amplifying inflammatory cascades and consequently Aβ and tau accumulation (Parhizkar and Arzberger 2019; Haass 2021). Together, these findings indicate that dysregulated cholesterol homeostasis amplifies innate immune activation through convergent TLR–TREM2–inflammasome signaling, establishing a chronic proinflammatory milieu in AD.

#### 4.5.3 Cerebrovascular Injury and White Matter Pathology

The effects of cholesterol imbalance extend beyond neurons and glial to the cerebrovascular system, which is commonly impacted in patients with AD (Fisher et al. 2022). Elevated levels of 27‐HOC, 25‐HOC, and 7‐KC have been shown to impair endothelial cell function, increase BBB permeability, and promote vascular inflammation (Anderson and Campo 2020; Umetani and Ghosh 2014; Tapia and Withers 2025). Oxysterol‐induced oxidative stress additionally triggers endothelial apoptosis and tight‐junction disruption (Gargiulo et al. 2016), contributing to vascular rarefaction and perivascular space (PVS) enlargement (Gouveia‐Freitas and Bastos‐Leite 2021), hallmarks of neurovascular injury in AD.

These pathogenic mechanisms contribute to the development of white matter hyperintensities, microbleeds, and microinfarcts seen in both AD and cerebral small vessel disease. Because myelin is highly enriched in cholesterol, oligodendrocytes and myelin maintenance are particularly sensitive to disturbances in lipid homeostasis (Wuerch and Yong 2025). Impaired cholesterol turnover and lipid transport may therefore compromise oligodendrocyte function and myelin stability, providing an additional pathway linking cholesterol dyshomeostasis to white matter injury in AD. Elevated 25‐HOC amplifies ischemic injury by impairing mitochondrial respiration and promoting oxidative stress, while dysregulated 24S‐HOC not only reflects disrupted neuronal cholesterol turnover but also correlates with white matter hyperintensities (WMH) burden and altered vascular lipid flux (Hughes and Kuller 2012).

Together, the oxysterol‐driven collapse of vascular support systems establishes a permissive environment for the progression of AD. Endothelial inflammation and BBB breakdown reduce the brain's capacity to clear Aβ (Zhang et al. 2022). Concurrent neurovascular uncoupling limits metabolic support to vulnerable white matter and hippocampal circuits, increasing susceptibility to tau phosphorylation, axonal injury, and neurodegeneration (Park and Hochrainer 2020; Liu and Cheng 2021; Girouard and Iadecola 2006). Therefore, cholesterol‐related vascular injury does not necessarily represent a parallel comorbidity, but actively amplifies core AD pathologies by impairing Aβ clearance, heightening oxidative stress, and accelerating tau‐related degeneration.

## 5 Therapeutic Strategies

Growing evidence that cholesterol imbalance contributes to synaptic dysfunction, neuroinflammation, vascular injury, and amyloidogenic processing has positioned lipid metabolism as an attractive therapeutic frontier in AD. Although current treatments largely target downstream features of neurodegeneration, such as amyloid and tau pathology, emerging approaches are increasingly directed toward correcting upstream disruptions in cholesterol synthesis, metabolism, transport, and lipid homeostasis. This section outlines existing therapeutic strategies and highlights future opportunities for selectively restoring central cholesterol balance in AD (Table 1).

**TABLE 1:** Summary of therapeutic strategies that target cholesterol homeostasis.

Drug	Target	Mechanistic category	Evidence level for impact on cholesterol and AD‐related outcomes	Concerns
*Cholesterol synthesis*				
Statins	HMGCR	Cholesterol synthesis inhibition (presqualene pathway)	Large observational evidence; mixed RCT findings for AD	High‐dose toxicity (rhabdomyolysis)
Squalene synthase inhibitors	SQS/FDFT1	Cholesterol synthesis inhibition (presqualene pathway)	Preclinical evidence for reduced Abeta; no active clinical development	Hepatotoxicity
Squalene epoxidase inhibitors	SQLE	Cholesterol synthesis inhibition (postsqualene pathway)	Preclinical cholesterol‐lowering evidence	No‐AD focused data
DHCR24 enhancers	DHCR24	Cholesterol synthesis (astrocytic postsqualene pathway)	Strong preclinical rationale	No drug candidates
*Cholesterol metabolism*				
Efavirenz	CYP46A1	Central cholesterol metabolism	Preclinical evidence; pilot human studies; ongoing phase 2 trial	Potential neuropsychiatric effects at higher doses
*Cholesterol transport*				
LXR agonists	LXR	Cholesterol transport	Robust preclinical synaptic and lipidation effects	Limited CNS‐selective agents
*Other*				
N‐Acetylcysteine (NAC)	Glutathione	Oxidative stress reduction	Preclinical and phase 1/2 clinical trials	Unknown effects on cholesterol and lipid pathways
Low‐dose IL‐2	Regulatory T cell expansion	Inflammation	Phase 1/2 trials in AD	Unknown effects on cholesterol and lipid pathways
sEH inhibitors	sEH	PUFA epoxide preservation; reduced diol formation; indirect effects on cholesterol homeostasis	Preclinical evidence; early human safety data in non‐AD participants	No AD trials to date; P‐domain selective inhibitors not yet available

### 5.1 Cholesterol Synthesis

HMGCR, the rate limiting enzyme of the presqualene pathway, is the principal target of statins, widely used to reduce systemic cholesterol levels. However, their central effects may depend on lipophilicity. Specifically, lipophilic statins (e.g., simvastatin, atorvastatin) penetrate the BBB more readily than hydrophilic statins (e.g., pravastatin, rosuvastatin), raising questions about their potential neuroprotective potential. Notably, a meta‐analysis of observational studies with more than 7 million patients reported that lipophilic statins demonstrated slightly greater protection against AD risk compared to hydrophilic (Westphal Filho and Moss Lopes 2025). However, the benefit of statins on dementia risk has not been replicated by other randomized controlled trials, highlighting the need for more robust trials with biological and clinical endpoints to determine whether statins confer a biological and/or clinical benefit on AD‐related outcomes. Though the lack of efficacy for AD‐related outcomes in randomized controlled trials may rationalize the need for trials with higher doses, it is important to note that high doses of statins carry risks such as rhabdomyolysis, underscoring the need for alternative therapeutic strategies that modulate cholesterol homeostasis more selectively within the brain. Inhibitors of squalene synthase (SQS/FDFT1), the enzyme catalyzing FPP to squalene, have garnered interest as an alternative treatment targeting the presqualene pathway, and have been investigated for the management of hyperlipidemia and atherosclerosis (Menys and Durrington 2003; Tavridou and Manolopoulos 2009; Davidson 2007). Preclinical studies have demonstrated that squalene synthase inhibitors reduce cholesterol levels and shift APP processing toward non‐amyloidgenic pathways, lowering Ab production (Menys and Durrington 2003; Bate and Williams 2007). However, squalene synthase inhibitors are not currently approved for therapeutic use due to safety concerns such as hepatotoxicity (Elsayed and Evans 2008). Nonetheless, targeting downstream or parallel nodes of the cholesterol synthesis pathway may offer alternative strategies that are safer and more precise in restoring cholesterol homeostasis in AD.

Although therapeutic interventions targeting the post‐squalene pathway are not yet approved, potential targets are being actively investigated. Inhibitors of SQLE, responsible for converting squalene, have been proposed as a strategy for lowering cholesterol (Padyana and Gross 2019), however, its impact on AD‐related outcomes has not been investigated. Enhancing astrocytic DHCR24 activity could offer another therapeutic approach by improving cholesterol supply to neurons, supporting synaptic maintenance, while stabilizing neuronal DHCR7 function might reduce oxidative stress from 7‐DHC accumulation. Such strategies could complement existing cholesterol‐targeted approaches, including statins or squalene pathway modulators, offering a more precise and potentially safer avenue for restoring cholesterol homeostasis in AD.

Targeting regulators of the cholesterol synthesis pathway may offer an alternative therapeutic strategy with potential benefits on AD pathogenesis. Specifically, agents that enhance SREBP2–DHCR24 signaling or restore astrocytic cholesterol export could counteract metabolic vulnerability in AD. Selective activation of LXRs has shown potential to increase ApoE‐mediated cholesterol efflux and improve neuronal repair, though peripheral lipid effects remain a concern (Richartz and Yam 2025; Kirchgessner and Sleph 2016; Fessler 2018). Finally, interventions that fine‐tune these pathways in astrocytes and microglia, rather than systemic lipid lowering, may provide the most effective and brain‐selective therapeutic benefit.

### 5.2 Cholesterol Metabolism

Low‐dose efavirenz, a CYP46A1 activator, which is currently approved as an anti‐viral drug for human immunodeficiency virus (HIV), has been investigated in preclinical models and pilot trials with human participants, and has demonstrated target engagement through an increase in 24S‐HOC (Lerner and Arnold 2022; Petrov et al. 2019). Based on those findings, a phase 2 trial is currently ongoing with Efaviernz to investigate its clinical and biological efficacy on AD [EUCT: 2023‐509613‐37‐01]. This strategy highlights the potential of selecting enhancing brain cholesterol clearance as a means to restore cholesterol homeostasis and reduce AD pathogenesis.

While the efficacy of statins on AD pathogenesis is unclear, one study investigated the impact of statins on 24S‐HOC and demonstrated greater decreases with lipophilic statins (lovastatin and simvastatin) than with hydrophilic statins (pramvastatin) (Vega and Weiner 2003, 2007). However, additional comprehensive studies that account for statin lipophilicity, lipoprotein carriage, and subsequent impacts on AD‐related outcomes are needed to confirm the robustness of these findings.

### 5.3 Cholesterol Transport

Although direct pharmacological modulation of ABC transporters or APOE is not yet feasible, genetic variation in ABCA1, ABCA7, APOE, and LRP1 may serve as stratification tools in clinical trials, identifying individuals with disrupted cholesterol trafficking who may benefit from cholesterol‐modulating interventions. Theoretically, enhancing ABCA1/ABCG1‐mediated efflux, promoting ApoE lipidation, or upregulating LRP1 uptake could restore neuronal cholesterol supply, improve synaptic repair, and reduce glial inflammation.

### 5.4 Alternative Strategies

While interventions targeting cholesterol synthesis, metabolism, and transport are critical, additional upstream mechanisms, such as oxidative stress and inflammatory signaling, offer complementary therapeutic opportunities.

Oxidative stress and inflammation are both drivers and downstream consequences of cholesterol dyshomeostasis. Therefore, oxidative stress reduction and anti‐inflammatory strategies may help restore cholesterol homeostasis and slow AD progression. Preclinical and early clinical studies in AD have investigated agents such as N‐acetylcysteine (a glutathione precursor) (Tardiolo et al. 2018) and low‐dose IL2 (Faridar and Gamez 2025), which target oxidative stress and inflammation, respectively. However, those studies do not report on measures of cholesterol homeostasis, and therefore their respective impacts on cholesterol dyshomeostasis remain unclear.

sEH is a bifunctional enzyme that integrates cholesterol and PUFA metabolism, converting vasoprotective and anti‐inflammatory epoxides into less active or cytotoxic diols (Newman et al. 2003). Through increased diol formation, elevated sEH activity amplifies oxidative stress and inflammatory signaling, which in turn perturbs sterol‐sensitive regulatory pathways, including SREBP2‐dependent control of cholesterol synthesis and membrane lipid remodeling. Inhibition of sEH, particularly the hydrolase domain, has emerged as a compelling strategy to preserve protective epoxides, reduce oxidative stress, and attenuate cerebrovascular injury (Griñán‐Ferré et al. 2020; Minaz et al. 2018; Xu and Li 2006; Yang et al. 2020; Tang and Border 2024). Through the phosphatase domain of sEH, cholesterol homeostasis could be impacted intracellularly through modulation of intracellular sterol‐sensitive signaling pathways that regulate ER cholesterol sensing and SREBP2 processing, thereby influencing downstream expression of cholesterol biosynthetic enzymes. Although selective inhibitors of the phosphatase domain of sEH, which could more directly target cholesterol homeostasis through cholesterol synthesis, are not yet under active clinical investigation, preclinical studies suggest that targeting sEH may simultaneously improve membrane lipid balance, reduce glial activation, and enhance neurovascular function (Kramer and Woltersdorf 2019; Jono and Shinouchi 2026). By mitigating the downstream consequences of lipid dysregulation, sEH inhibition may complement interventions aimed at cholesterol synthesis, metabolism, and transport.

Given the complex interplay between cholesterol dysregulation, oxidative stress, neuroinflammation, and PUFA metabolism, combination strategies may offer the greatest therapeutic potential. For example, coupling sEH inhibition with selective modulation of cholesterol synthesis or metabolism could simultaneously stabilize membrane composition, restore cholesterol flux, and preserve anti‐inflammatory lipid mediators. Integrating antioxidant or anti‐inflammatory interventions alongside cholesterol‐targeted therapies may further enhance neuronal resilience and vascular integrity. These multi‐target approaches recognize the interconnected nature of lipid homeostasis in AD and highlight opportunities to optimize intervention timing, maximize efficacy, and tailor strategies to individual molecular profiles.

Ultimately, these strategies have the potential to mitigate multiple core features of AD simultaneously. By restoring cholesterol balance and reducing lipid‐mediated stress, they may improve amyloid clearance, limit tau hyperphosphorylation, and protect cerebrovascular integrity, thereby slowing or preventing white matter injury, neurodegeneration, and cognitive decline.

## 6 Conclusions

Cholesterol homeostasis in the brain is a finely tuned system that supports neuronal integrity, synaptic function, glial activity, and cerebrovascular health. Disruption of synthesis, metabolism, or transport, driven by aging, peripheral hypercholesterolemia, neurodegeneration, oxidative stress, and inflammation, creates a permissive environment for amyloidogenic processing, tau pathology, neuroinflammation, and vascular injury, all central to AD pathogenesis. Therapeutic strategies that selectively restore cholesterol homeostasis offer opportunities to intervene upstream in these pathological cascades. Complementary approaches targeting oxidative stress, inflammation, and PUFA metabolism, such as sEH inhibition, may further enhance neuronal and vascular resilience. Together, these insights underscore the potential of integrated, multi‐target interventions to stabilize lipid homeostasis, preserve cognitive function, and slow disease progression, highlighting cholesterol regulation as a critical axis for future research and therapeutic development in AD.

## Author Contributions

**Myuri Ruthirakuhan:** conceptualization, writing – original draft, visualization, writing – review and editing, investigation. **Walter Swardfager:** conceptualization, writing – review and editing, supervision. **Ameer Y. Taha:** writing – review and editing.

## Funding

Dr. Ruthirakuhan is funded by a Canadian Institutes of Health Research—Research Excellence, Diversity and Independence (CIHR‐REDI) Award (ECA‐508989). Dr. Taha is funded by NIA/NIH R56AG083336 and the USDA National Institute of Food and Agriculture, Hatch/Taha (project #1008787). Dr. Swardfager is funded by the Canada Research Chairs Program (Award Number: CRC‐2024‐00213), the Canadian Institutes of Health Research (PJT‐159711), the Ontario Ministry of Colleges and Universities (Award Number: ER21‐16‐146), and the Dr. Sandra Black Centre for Brain Resilience and Recovery.

## Conflicts of Interest

The authors declare no conflicts of interest.

## Data Availability

Data sharing not applicable to this article as no datasets were generated or analyzed during the current study.
